# Microbial structure and nitrogen compound conversions in aerobic granular sludge reactors with non-aeration phases and acetate pulse feeding

**DOI:** 10.1007/s11356-016-7709-7

**Published:** 2016-09-23

**Authors:** Agnieszka Cydzik-Kwiatkowska, Paulina Rusanowska, Magdalena Zielińska, Katarzyna Bernat, Irena Wojnowska-Baryła

**Affiliations:** University of Warmia and Mazury in Olsztyn, Słoneczna 45G, 10-709 Olsztyn, Poland

**Keywords:** *nosZ*, *nirK*, *nirS*, *amoA*, Anammox bacteria, Aerobic granules, Anoxic phases, Low COD/N

## Abstract

A technological system was developed for efficient nitrogen removal from real digester supernatant in a single reactor with shortened aeration to increase the economical aspects of wastewater treatment. The supernatant (600 mg TKN/L, low COD/N ratio of 2.2) was treated in batch reactors with aerobic granules (GSBRs) to test how one, two, or three non-aeration phases and acetate pulse feeding in the cycle affect the morphological and microbial properties of biomass. Introduction of one non-aeration phase in the cycle increased nitrogen removal efficiency by 11 % in comparison with constantly aerated GSBR. The additional non-aeration phases did not diminish the efficiency of ammonia oxidation but did favor nitrification to nitrate. Acetate pulse feeding in the reactor with three non-aeration phases raised the efficiency of nitrogen removal to 77 %; in parallel, the number of denitrifiers possessing *nosZ* genes and performing denitrification to N_2_ increased. Ammonia was oxidized by aerobic and anaerobic ammonia-oxidizing bacteria and heterotrophic nitrifiers (*Pseudomonas* sp. and *Alcaligenes faecalis*) that coexisted in granules. *Azoarcus* sp., *Rhizobium* sp., and *Thauera* sp. were core genera of denitrifiers in granules. An increase in the number of non-aeration phases diminished EPS content in the biomass and granule diameters and increased granule density.

**Electronic supplementary material:**

The online version of this article (doi:10.1007/s11356-016-7709-7) contains supplementary material, which is available to authorized users.

## Introduction

Purification of wastewater with high concentrations of nitrogen and an unfavorable ratio of organic compounds to nitrogen (COD/N) is a current problem in wastewater treatment. The use of activated sludge technology often results in only partial ammonia removal from high-ammonia wastewater streams (Bernat et al. 2012). This disadvantage can be overcome by the use of aerobic granular sludge systems. In the reactors with aerobic granular sludge, efficient N removal of 60–70 % was observed from wastewater with a high concentration of ammonia nitrogen (from 400 to 2000 mg/L) and a high COD/N ratio (3.9–6.9) (Yu et al. 2014). For high-nitrogen wastewater (569 mg TKN/L) characterized by a low COD/N ratio of 1.4 treated in granule sequencing batch reactors (GSBRs) with continuous aeration, full ammonia oxidation was easily obtained, but the overall efficiency of nitrogen removal was much lower (Cydzik-Kwiatkowska et al. 2013). When using aerobic granular sludge to purify wastewater of a low COD/N ratio and high-ammonia concentration, nitrogen removal efficiency may be improved by adjusting the reactor cycle by introducing alternating aeration/non-aeration phases and/or adding external organics during the reactor cycle. Alternating oxic/anoxic conditions induce a diauxic phase in bacterial cells which enables the resynthesis of denitrification enzymes and supports N removal (Lee et al. 2010). The benefit of these conditions for wastewater treatment efficiency was shown in GSBRs treating wastewater at a low nitrogen load (Zhang et al. 2011).

In aerobic granules, diverse heterotrophic and autotrophic nitrogen-converting microorganisms are active (Vlaeminck et al. 2008; Cydzik-Kwiatkowska and Wojnowska-Baryła 2015). Their abundance and species composition influence the efficiency and stability of nitrogenous compound removal. Most research focuses on the microorganisms involved in ammonia oxidization, which is a crucial step for nitrogen removal. Heterotrophic denitrifiers are, however, less well studied because they belong to many different phylogenetic groups, and denitrification can be easily obtained in most wastewater treatment systems with anoxic tanks. A problem occurs when the treatment of high-ammonia wastewater is carried out in a single reactor. In these one-step systems, the process is mainly designed for efficient ammonia oxidation which requires aeration and low COD levels in the reactor. Aerobic granular sludge systems partially overcome this problem. Due to diffusion limits in granule structure, simultaneous nitrification and denitrification may occur in biomass despite the aeration of the reactor. In these systems, little is known about the impact of reactor working cycle modifications on nitrogen removal by heterotrophic, autotrophic, and aerobic denitrifiers. The species composition of denitrifiers in biomass is determined, among other things, by the COD/N ratio, the type of organic carbon, and the reactor setup (Wan et al. 2011). Not all denitrifying bacteria carry out complete denitrification. Some bacteria may be missing nitrate reductase, others, nitrous oxide reductase, but by growing in complex communities, the metabolic processes of each species can be complemented by the processes of another species. For complete denitrification to N_2_, it is important to control the process because the final enzyme in this pathway (dinitrogen oxide reductase) is sensitive to the presence of oxygen, nitrite, and large amounts of process intermediates (Kampschreur et al. 2009). Very efficient reduction of nitrate and minimal formation of intermediate products have been obtained with the use of acetate as a carbon source in pure culture of *Pseudomonas stutzeri* D6 (Yang et al. 2012).

Modifications in the GSBR cycle influence the morphology and physicochemical characteristics of granules, which influences nitrogen conversions within the multilayered structure of the granules. Small granules (0.5–0.9 mm) have greater activity of ammonia-oxidizing bacteria because oxygen penetrates the granule more easily (Wang et al. 2014a), whereas larger granules (>1.3 mm) have a larger anoxic zone that favors denitrification (Li et al. 2012). The structure of granules is the result of a network of connections between individual cells and extracellular polymeric substances (EPS) produced by bacteria (Sheng et al. 2010). EPS create a layer that buffers microorganisms against the unfavorable environment, favors organic pollutant absorption, and provides a carbon and energy source during bacterial starvation (Flemming and Leis 2002). As previously shown, both the GSBR cycle length and the COD/N ratio in the influent affect EPS production (Cydzik-Kwiatkowska et al. 2014).

The study explored interrelationships between the introduction of non-aeration periods and organics dosage and community composition and nitrogen removal efficiency in the GSBRs treating high-nitrogen digester supernatant. The technological research was supported by a broad range of molecular analyses that enabled conclusions to be drawn about the ecology, microbial composition, and EPS production in complex biocenoses of aerobic granules. Investigations of functional genes were used to determine which metabolic pathways were promoted by the operational conditions and which microorganisms were involved in nitrogen conversions. A technological solution was devised for efficient nitrogen removal from real digester supernatant in a single reactor with diminished aeration that increases the economical aspects of wastewater treatment.

## Materials and methods

### Substrate

Digester supernatant from dewatering of anaerobically stabilized sewage sludge in the municipal wastewater treatment plant in Olsztyn (Poland) was the influent for the experimental reactors. The average pollutant concentrations in the influent were 1350 ± 145 mg COD/L, 600 ± 32 mg TKN/L, 493 ± 21 mg NH_4_-N/L, and 12 ± 3.2 mg P/L; COD/N ratio was 2.2, and the BOD_5_/COD ratio was 0.6. Carbonates and bicarbonates were added to the digester supernatant in stoichiometric amounts to maintain alkalinity for nitrification. pH of digester supernatant was 8.5, and alkalinity was 70 mval/L.

### GSBR operation

The experiment was carried out in four column GSBRs with a working volume of 4.5 L, height 57 cm, and diameter 10 cm, which were operated by programmable logic controllers at a volumetric exchange rate of 60 %, a temperature of 26 °C, and a pH of 7.5–8.5. Air was supplied continuously at a rate of 4 L/min (superficial gas velocity of 0.85 cm/s). The cycle length was 8 h (hydraulic retention time of 13 h) and consisted of 5 min of filling, 465 min of reaction phase, 5 min of settling, and 5 min of decantation. The GSBRs differed in the number of 30-min non-aeration phases in the reaction phase. The lengths of phases in the GSBR cycles are given in Table 1. Mixing in non-aeration phases was provided by mechanical stirrers (stirrer diameter 7 cm); the mixing intensity was 0.016 g. *R*_3+ac_ was operated as *R*_3_, but at the beginning of the second non-aeration phase, sodium acetate was pulse-fed at a dose of 500 mg COD/L. The ratio of total organic compounds introduced to the *R*_3+ac_ twice in the cycle (in the influent and as an acetate pulse feeding in the second non-aeration phase) to the amount of nitrogen in the influent was 3.0. The sludge retention time (SRT) was similar and varied between 7 and 10 days thus showing comparable conditions in the GSBRs. In all reactors, at least 400 cycles were conducted (133 days of the GSBR operation).

**Table 1:** The lengths of phases in the GSBR cycle

Phases in the GSBR cycle		*R*_0_	*R*_1_	*R*_2_	*R*_3_	*R*_3+ac_
Filling (min)		5	5	5	5	5
Reaction phase	Non-aeration I (min)	−	30	30	30	30
	Aeration I (min)	435	435	120	120	120
	Non-aeration II (min)	−	−	30	30	30
	Aeration II (min)	−	−	285	120	120
	Non-aeration III (min)	−	−	−	30	30
	Aeration III (min)	−	−	−	135	135
Sedimentation (min)		5	5	5	5	5
Decantation (min)		5	5	5	5	5

As a seed sludge, aerobic granules were taken from a control reactor with constant aeration in the reaction phase (*R*_0_) that was fed with high-nitrogen digester supernatant (Cydzik-Kwiatkowska et al. 2013). In *R*_0_, biomass concentration was 6.4 ± 1.5 g MLSS/L and granule diameter was 0.91 ± 0.20 mm. Ammonia was fully removed; denitrification efficiency was 17.5 ± 7.9 %, and COD removal was 59 ± 19 %. In the effluent, the concentration of oxidized nitrogen forms was ca. 400 mg/L; nitrite made up 90 % of this concentration.

### Physicochemical analyses

Concentrations of ammonia, nitrite, nitrate, total Kjeldahl nitrogen (TKN), total phosphorus, and COD in the GSBR influent and effluent, the biomass settling properties (sludge volumetric index (SVI)), and biomass concentration in the GSBRs (g MLSS/L, g MLVSS/L) and in the effluent (g TSS/L) were analyzed in accordance with APHA (1992). Nitrate, nitrite, and ammonia concentrations were measured as mg NO_3_-N/L, mg NO_2_-N/L, and mg NH_4_-N/L. pH and alkalinity were measured by using TitroLine Easy (SI Analytics). At the end of the experimental period, all these physicochemical analyses were carried out during the GSBR cycles to observe the changes in pollutant concentrations over time. The changes in ammonia, nitrite, and nitrate concentrations followed zeroth-order kinetic, while COD changes followed pseudo-first-order kinetic. To calculate nitrification efficiency, the concentration of the oxidized nitrogen forms was divided by the difference between TKN concentration in the reactor and in the effluent less the N used for biomass synthesis. The concentration of N used for biomass synthesis was calculated as multiplication of sludge yield coefficient, concentration of COD removed, and percentage of N in the biomass. The N percentage in the biomass (N%) was calculated as follows: at the end of the cycle, a mixture of biomass and wastewater was sampled from the reactor, TKN was measured in this mixed sample (1), and in this sample after filtration through 0.45-μm filter (2), the subtraction of TKN from samples 1 and 2 was divided by the biomass concentration in sample 1. The obtained result was in mg N/mg MLSS, and it was expressed as N%. For denitrification efficiency, the concentration of N reduced was divided by the concentration of all oxidized nitrogen forms. For these calculations, the results from the last 200 cycles of stable reactor performance were used. Dissolved oxygen (DO) concentration was measured by using a ProODO probe (YSI Environmental). The morphology of the granules in each reactor was determined at the end of the experimental period on the basis of free settling as described by Cydzik-Kwiatkowska et al. (2009). Photos were taken with an OLYMPUS camera with a resolution of 10 Mpics. Two hundred and five of the photographed granules were analyzed by using the Image Tool Version 3.0 (Wilcox et al. 2002). The amount of EPS was measured according to Hang et al. (1999) and expressed per a unit of COD removed (g EPS/g COD_rem_) as described by Laspidou and Rittmann (2002). Relative hydrophobicity was measured in accordance with Chang and Lee (1998).

### Molecular methods

#### DNA isolation

Biomass from each GSBR was sampled twice at the end of the experiment and frozen at −20 °C prior to molecular analysis. From each biomass replicate, DNA was extracted from approximately 400 mg of centrifuged sample by using a FastDNA® SPIN®Kit (Q-BIOgene). The DNA from both isolations was mixed. The concentration of DNA was measured spectrophotometrically by using a BioPhotometer (Eppendorf), and working solutions with a DNA concentration of 50 ng/μL were prepared.

#### PCR-denaturant gradient gel electrophoresis

To assess the species diversity of the microorganisms in granules from the experimental reactors, polymerase chain reaction-denaturant gradient gel electrophoresis (PCR-DGGE) was used. Four primer sets were applied that recognized the sequences of the *amoA* gene coding for ammonia monooxygenase (the first step of nitrification), the *nirK* and *nirS* genes coding for nitrite reductase containing Cu or *cd1* cytochrome, respectively (reduction of nitrite to nitric oxide), and the *nosZ* gene coding for nitrous oxide reductase (reduction of nitrous oxide to molecular nitrogen) (Table 2). PCR was performed in an Eppendorf® Mastercycler Gradient (Eppendorf). The PCR mixture contained 1.7 ng/μL of extracted DNA, 0.5 μM of each primer, 100 μM of deoxynucleoside triphosphate mixture (Promega), 0.05 U/μL of GoTaq® DNA Polymerase (Promega), 6 μL of 10× reaction buffer supplied with polymerase, 1.5 mM MgCl_2_, and sterile water for a final volume of 30 μL. The details of amplification and DGGE of the *amoA* gene and the denitrification genes *nirK*, *nirS*, and *nosZ* are given in Cydzik-Kwiatkowska and Wojnowska-Baryła (2011) and Cydzik-Kwiatkowska et al. (2014), respectively. PCR-DGGE was repeated at least three times for each sample. Clear and intense bands were excised from the DGGE gel, reamplified, and sequenced at the Institute of Biochemistry and Biophysics of the Polish Academy of Science (http://www.oligo.ibb.waw.pl). The nucleotide sequences were compared with the sequences in FunGene and GenBank by using the BLASTn program and then aligned by the maximum likelihood method with the use of MEGA5 software (Tamura et al. 2011). The sequences identified in the study were deposited in the GenBank under accession nos. KF738710-KF738752, KP858121-KP858141, and KT822442-KT822448.

**Table 2:** Primers used in PCR-DGGE and real-time PCR and conditions applied in real-time PCR

Gene, primer	Technique	Reference	Primer conc.	Thermal profile	Reference
*amoA*, amoA1F^a^/amoA2R	Real-time PCR, PCR-DGGE	Rotthauwe et al. (1997)	100 nM	94 °C, 15 s 52 °C, 45 s 60 °C, 45 s	Cydzik-Kwiatkowska and Wojnowska-Baryła (2015)
Bacterial 16S rDNA, 519F/907R	Real-time PCR	Lane (1991)	100 nM	94 °C, 15 s 50 °C, 40 s 60 °C, 40 s	Cydzik-Kwiatkowska and Wojnowska-Baryła (2015)
16S rDNA of Anammox bacteria, amx809F/amx1066R	Real-time PCR	Tsushima et al. (2007)	100 nM	94 °C, 15 s 60 °C, 1 min	Cydzik-Kwiatkowska et al. (2014)
*nirK*, F1aCu/R3Cu^a^	Real-time PCR, PCR-DGGE	Throbäck et al. (2004)	150 nM	94 °C, 15 s 55 °C, 1 min, 60 °C, 1 min	Cydzik-Kwiatkowska et al. (2014)
*nirS*, cd3aF/R3cd^a^	Real-time PCR, PCR-DGGE		150 nM	94 °C, 15 s 53 °C, 1 min, 60 °C, 1 min	
*nosZ*, NosZ-F /NosZ1622R^a^	Real-time PCR, PCR-DGGE	Kloos et al. (2001), Throbäck et al. (2004)	200 nM	94 °C, 15 s 60 °C, 2.5 min	Cydzik-Kwiatkowska et al. (2014)

^a^The primer contains a 33-bp GC clamp (5′ GGC GGC GCG CCG CCC GCC CCG CCC CCG TCG CCC 3′)

#### Relative real-time PCR

Real-time PCR was performed to assess the relative abundance of total bacteria, ammonia-oxidizing bacteria (AOB), denitrifiers, and Anammox bacteria in the aerobic granules. A reaction mixture contained 3 ng/μL of the template DNA, 12.5 μL of Maxima SYBR Green/ROX qPCR Master Mix (2×) (Thermo Scientific), primers (the concentration of each primer is presented in Table 2), and water for a final volume of 20 μL. The amplification reaction started with 2 min at 50 °C and 10 min at 95 °C; then, 40 cycles of amplification proceeded according to the thermal profiles described in Table 2. During *amoA* gene and *nosZ* gene amplifications, 50 and 25 nM of KCl, respectively, were added to increase the reaction specificity. Each DNA sample was amplified in triplicate in the presence of negative and positive controls. The amplifications were followed by a dissociation step to confirm the melting temperature of the PCR products, and the products were also electrophoresed in the presence of a molecular marker. Reactions were carried out in a 7500 Real-Time PCR System (Applied Biosystems). Data were analyzed with Sequence Detection Software, version 1.3 (Applied Biosystems). The relative abundance of the investigated genes in the granules was compared by using a modification of the 2^−ΔΔCt^ method (Livak and Schmittgen 2001). Reactions were normalized by adding the same amount of DNA for each reaction tube. Seed sludge from constantly aerated *R*_0_ was used as a calibration sample.

### Calculation methods

To gain insight into how the operational conditions of treatment affect the composition of bacteria possessing *amoA*, *nirK*, *nirS*, and *nosZ* genes, canonical correspondence analysis (CCA) was performed (including reactor *R*_0_) (ter Braak and Smilauer 2002) on the DGGE band intensities with Monte Carlo permutation testing (expressed in F statistics, 499 permutations). To the CCA, metadata obtained by DGGE and environmental variables, i.e., the number of non-aeration phases in the cycle (nap), the presence of external organics in the non-aeration phase, and the concentrations of nitrate and nitrite in the effluent, were taken. These analyses were carried out by using CANOCO for Windows ver. 5.0 (Microcomputer Power). The diversity of different bacterial groups was assessed on the basis of the number and intensity of DGGE bands in an electrophoretic lane and expressed with the Shannon-Wiener index of diversity (*H*′).

The correlations between the groups of the results were analyzed by using a Pearson’s correlation with Statistica 10.0 (StatSoft); the strength and direction of correlation were expressed by correlation coefficient (*R*). Differences between two samples were tested for significance by using a *t* test for independent samples. To compare more than two samples, ANOVA and the Tukey test (*t*—value of the test) were used after the normality and homogeneity of variance were confirmed with the Shapiro-Wilk test and Levene’s test. In the cases of non-normal distribution, the non-parametric Kruskall-Wallis test was used. With all tests, *p* < 0.05 was considered significant.

## Results

### Technological results

**Fig. 1:**
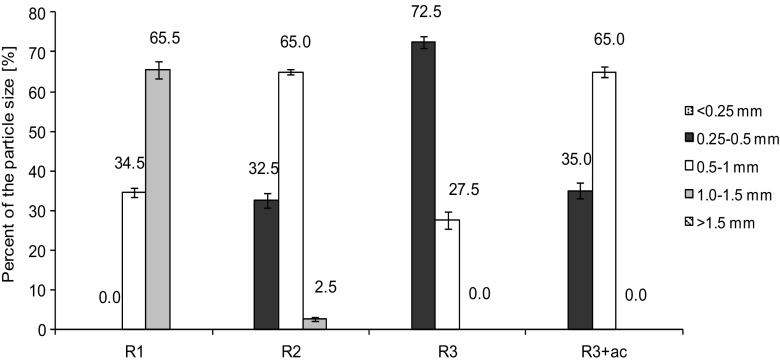
The distribution of granule particle sizes

The concentration of granular sludge was stable in each GSBR and decreased with an increase in the number of non-aeration phases from 10.8 ± 2.2 g MLSS/L in *R*_1_ to 8.3 ± 2.5 and 6.6 ± 1.5 g MLSS/L in *R*_2_ and *R*_3_, respectively. In *R*_3+ac_, biomass concentration increased to 9.9 ± 3.1 mg MLSS/L. The organic fraction of the biomass ranged from 62.5 to 69.5 % in *R*_2_ and *R*_1_, respectively, indicating a high quality of aerobic granules. The concentrations of MLSS in the effluents were 0.36 ± 0.06, 0.37 ± 0.09, 0.46 ± 0.22, and 0.38 ± 0.06 g TSS/L in *R*_1_, *R*_2_, *R*_3_, and *R*_3+ac_, respectively. The time courses of DO showed that it declined to 0 mg/L during the first non-aeration phase in *R*_1_ and the first and the second non-aeration phases in *R*_2_, *R*_3_, and *R*_3+ac_ (Fig. 1SM). After 4–5 h of the cycle, ammonia and easily biodegradable COD were oxidized; therefore, DO was not fully depleted in the third non-aeration phase and only decreased to about 2–3 mg/L. DO reached at least 6 mg/L by the end of the GSBR cycles.

In *R*_1_, granules had the largest average diameter of approx. 1.1 mm, the highest weight (0.00223 ± 0.0014 mg), and the highest settling velocity of 1.70 ± 1.06 mm/s (Table 3). In *R*_1_, granules with a diameter of 1.0–1.5 mm accounted for 65.5 % of all granules (Fig. 1). Introduction of non-aeration phases in the GSBR cycle reduced the mass of the granules, their diameters, and settling velocity (*R* = −0.98, *R* = −0.94, and *R* = −0.99, respectively). The average size of the granules was 0.59 ± 0.17 mm in *R*_2_ and 0.48 ± 0.11 mm in *R*_3_. The contribution of granules having a diameter in the range of 0.25–0.50 mm rose with the increase in the number of non-aeration phases in the GSBR cycle; these granules accounted for over 72 % of biomass in *R*_3_. The pulse feeding of acetate in *R*_3+ac_ increased the granule diameters to about 0.58 ± 0.16 mm which translated into their higher settling velocity (Table 3). In this GSBR, the biomass was predominated (65 %) by granules with diameters from 0.5 to 1 mm.

**Table 3:** Biomass characteristics in the GSBRs

	*R*_1_	*R*_2_	*R*_3_	*R*_3+ac_
Granule diameter (D) (mm)	1.10 ± 0.15	0.59 ± 0.17	0.48 ± 0.11	0.58 ± 0.16
Settling velocity (V) (mm/s)	1.70 ± 1.06	1.42 ± 1.56	0.96 ± 0.72	1.34 ± 0.82
V/D ratio (s^−1^)	1.56 ± 1.00	2.29 ± 2.00	2.06 ± 1.55	2.27 ± 1.01
Density of granule with water (*ρ*_e_) (kg/m^3^)	1002.77 ± 2.26	1007.98 ± 6.45	1010.26 ± 8.14	1009.07 ± 4.88
Mass of the granule (m) (mg)	0.00223 ± 0.0014	0.00115 ± 0.0015	0.00056 ± 0.0004	0.00102 ± 0.0009
Fractal dimension (F) (−)	2.87 ± 0.08	2.77 ± 0.09	2.75 ± 0.08	2.74 ± 0.06
Sludge volume index (SVI) (mL/g MLSS)	20.54 ± 2.12	30.60 ± 8.43	36.51 ± 10.74	32.42 ± 10.96
Hydrophobicity (%)	73.9 ± 7.60	46.6 ± 4.97	26.5 ± 7.94	28.6 ± 2.89
EPS (g/g MLSS)	0.34 ± 0.027	0.14 ± 0.02	0.17 ± 0.01	0.39 ± 0.04
EPS (g/g COD_rem_)	0.16 ± 0.01	0.06 ± 0.01	0.08 ± 0.01	0.17 ± 0.01

In general, an increase in granule diameter resulted in an increase in granule mass (*R* = 0.98) and fractal dimension (*R* = 0.97) and a decrease in their density (*R* = −0.99). In all GSBRs, the fractal dimension (*F*) was high pointing to spherical shape of granules and dense packing of microorganisms on their surface. SVI reflected the settling properties of the granules and was mostly related to the settling velocity of the biomass. In the present study, the lowest SVI of ca. 21 mL/g MLSS characterized the biomass from *R*_1_; in the reactors with at least two non-aeration phases, SVI was 1.5–1.8 times higher, but it still was very low thus indicating very well settling biomass. The settling velocity (according o Stoke’s law) depends on, among others, granule density, mass, and fractal dimension of the granules (Cydzik-Kwiatkowska et al. 2009).

The hydrophobicity of biomass, indicating its ability to aggregate, decreased with an increasing number of non-aeration phases in the GSBR cycle (*R* = −0.99). It was not affected by the pulse feeding of acetate. Hydrophobicity of granules was also positively correlated with their masses and fractal dimensions (*R* = 0.96, *R* = 0.97, respectively) and negatively correlated with granule densities (*R* = −0.98).

Introduction of non-aeration phases in the GSBR cycle decreased EPS content in the biomass both per gram of MLSS and per load of COD removed. EPS content in biomass in *R*_1_ averaged 0.34 ± 0.027 g/g MLSS and was lowered to 0.14 ± 0.02 and 0.17 ± 0.01 g/g MLSS in *R*_2_ and *R*_3_, respectively. The acetate pulse feeding in *R*_3+ac_ caused a significant increase in EPS content in the biomass, when compared to *R*_2_ and *R*_3_, both per gram of MLSS and the load of COD removed (respectively *t* = 0.008, *t* = 0.010, *t* = 0.015, and *t* = 0.041).

Organic and nitrogen loadings were 2.0 g COD/(L day) and 1.1 g TKN/(L day). The highest efficiency of COD removal (77.5 ± 7.2 %) was noted in *R*_3+ac_ (Table 4), and this was significantly higher than in *R*_3_. An increase in the number of non-aeration phases from 1 to 3 decreased the specific COD removal rate (Table 4). The COD removal rate in *R*_3+ac_ was 254.4 mg/(g MLVSS h) in the first non-aeration phase and lowered to 129.5 mg/(g MLVSS h) after the pulse feeding of acetate in the second non-aeration phase.

**Table 4:** Kinetic parameters of the organics and ammonia removal, nitrite and nitrate conversions, and effectiveness of nitrification, denitrification, and N and COD removal in the GSBRs

	*R*_1_	*R*_2_	*R*_*3*_	*R*_3+ac_
*k*_COD_ (h^−1^)	−6.3	−13.9	−4.1	−3.0/−3.2^a^
*r*_COD_ (mg/(g MLVSS h))	1538.0	1452.5	402.8	254.4 (2.5 h)/129.5 (5.15 h)^a^
*k*_NH4-N_ (mg/(L h))	−77.6	−77.1	−66.0	−65.0
*r*_NH4-N_ (mg/(g MLVSS h))	25.6	15.9	13.8	10.9
*k*_NO2-N_ (mg/(L h))	+ 22.4	−60.0/+71.2/−49.8	−175.8/+71.5/−57.8	−211.0/+56.4/−216.2/+69.2/−53.3
*r*_NO2-N_ (mg/(g MLVSS h))	7.2	11.5 (0.5 h)/14.7 (4 h)/10.3 (3.25 h)	36.8 (0.5 h)/15.0 (4.25 h)/12.1 (1.75 h)	35.3 (0.5 h)/9.4 (2 h)/36.2 (0.5 h)/11.6 (2.75 h)/8.9 (2 h)
*k*_NO3-N_ (mg/(L h))	11.0	−124.0/+17.0	−248.0/+15.4	0^b^/+26.2
*r*_NO3-N_ (mg/(g MLVSS h))	3.6	25.6 (0.5 h)/3.5 (7.25 h)	51.9 (0.5 h)/3.2 (7.25 h)	0^b^ (4 h)/4.4 (3.75 h)
Nitrification efficiency	93.7 ± 0.1	98.4 ± 6.2	96.4 ± 4.7	99.1 ± 2.9
Denitrification efficiency	24.1 ± 7.5	20.3 ± 4.0	22.8 ± 5.7	60.0 ± 7.6
N removal efficiency	35.5 ± 8.6	30.0 ± 5.1	32.9 ± 6.4	73.3 ± 5.9
COD removal efficiency	50.8 ± 16.6	69.6 ± 14.8	59.8 ± 11.9	77.5 ± 7.2

In *R*_2_, *R*_3_, and *R*_3+ac_, next to the constants of reaction rates, minus and plus mean a decline or an increase in the concentration; next to the reaction rates, the lengths of these decline or increase periods are given in brackets, for example, “*r*_NO2-N_ = 36.8 (0.5 h)” means that the nitrite decreased during half an hour at a rate of 36.8 mg/(g MLVSS h)

*k* constant of reaction rate, *r* reaction rate

^a^Values obtained after acetate pulse feeding

^b^Nitrate concentrations from 0.4 to 19.3 mg/L without clear changes

**Fig. 2:**
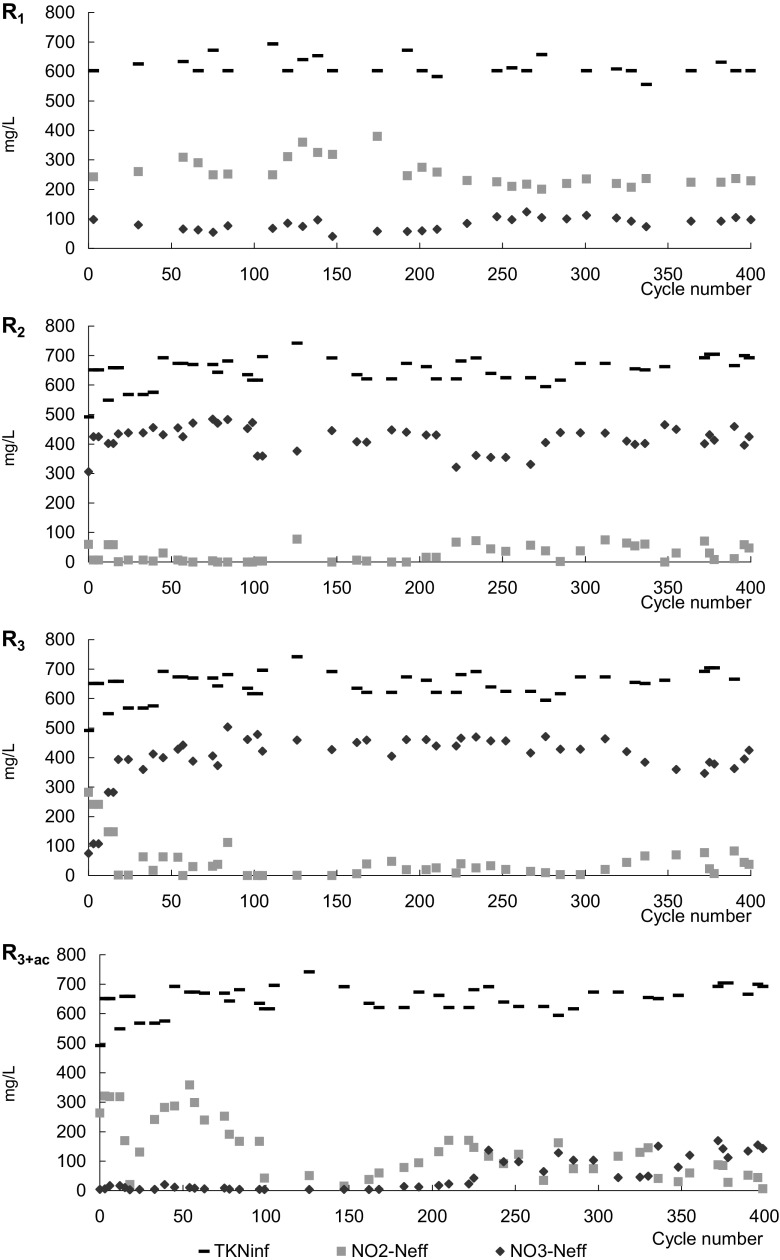
Concentration of total Kjeldahl nitrogen (TKN) in the influent and the concentrations of nitrite and nitrate in the effluent from the GSBRs

The efficiency of nitrification in all GSBRs was over 90 % (Table 4). The initial ammonia concentration in the cycle was about 320 mg NH_4_-N/L, and ammonia concentrations did not exceed 2.5 mg/L in any of the GSBR effluents. The introduction of one non-aeration phase in the cycle significantly increased denitrification efficiency to 24.1 ± 7.5 % in comparison to the constantly aerated control *R*_0_ (17.5 ± 7.9 %). Increasing the number of non-aeration phases in the cycle to 2 or 3 did not significantly influence either the efficiency of denitrification or nitrogen removal. Pulse feeding of acetate to *R*_3+ac_ significantly improved efficiencies of denitrification (60.0 ± 7.6 %) and nitrogen removal (73.3 ± 5.9 %) in comparison to the other experimental conditions. The use of ammonia for biomass synthesis in *R*_1_, *R*_2_, and *R*_3_ was about 20 mg/L and increased to 50.4 ± 4 mg/L in *R*_3+ac_. The average concentration of nitrite in the effluent from *R*_1_ (254.2 ± 50.2 mg/L) was significantly higher than in the other reactors (Fig. 2). Pulse feeding of acetate increased the average concentration of nitrite in the effluent to 90.5 ± 49.0 mg/L in *R*_3+ac_ in comparison with 43.2 ± 24.7 and 43.3 ± 28.2 mg/L in *R*_2_ and *R*_3_, respectively. The concentrations of nitrate in the effluents from *R*_2_ (425.0 ± 23.7 mg/L) and *R*_3_ (395.4 ± 35.4 mg/L) were significantly higher than in the effluents from *R*_1_ and *R*_3+ac_.

An increase in the number of non-aeration phases decreased the ammonia removal rate (*R* = −0.93). In *R*_1_, ammonia was completely oxidized in 4 h at a rate of 25.6 mg NH_4_-N/(g MLVSS h) (Fig. 1SM, Table 4) and the concentrations of nitrite and nitrate increased throughout the cycle at rates of 7.2 and 3.6 mg/(g MLVSS h), respectively. With at least two non-aeration phases in the cycle, the time required for complete removal of ammonia was about 5 h and the rate of ammonia removal ranged from 10.9 mg NH_4_-N/(g MLVSS h) in *R*_3+ac_ to 15.9 mg NH_4_-N/(g MLVSS h) in R_2_. In *R*_2_ and *R*_3_, the highest rates of nitrite and nitrate removal were observed in the first non-aeration phase. In *R*_3+ac_, nitrite was completely removed during the first non-aeration phase, and then, its concentration began to increase at a rate of 9.4 mg/(g MLVSS h). Pulse feeding of acetate resulted in complete reduction of nitrite. After this, nitrite once more increased to 202 mg/L at 11.6 mg/(g MLVSS h), then decreased at a rate of 8.9 mg/(g MLVSS h) (Table 4).

### Molecular results

**Fig. 3:**
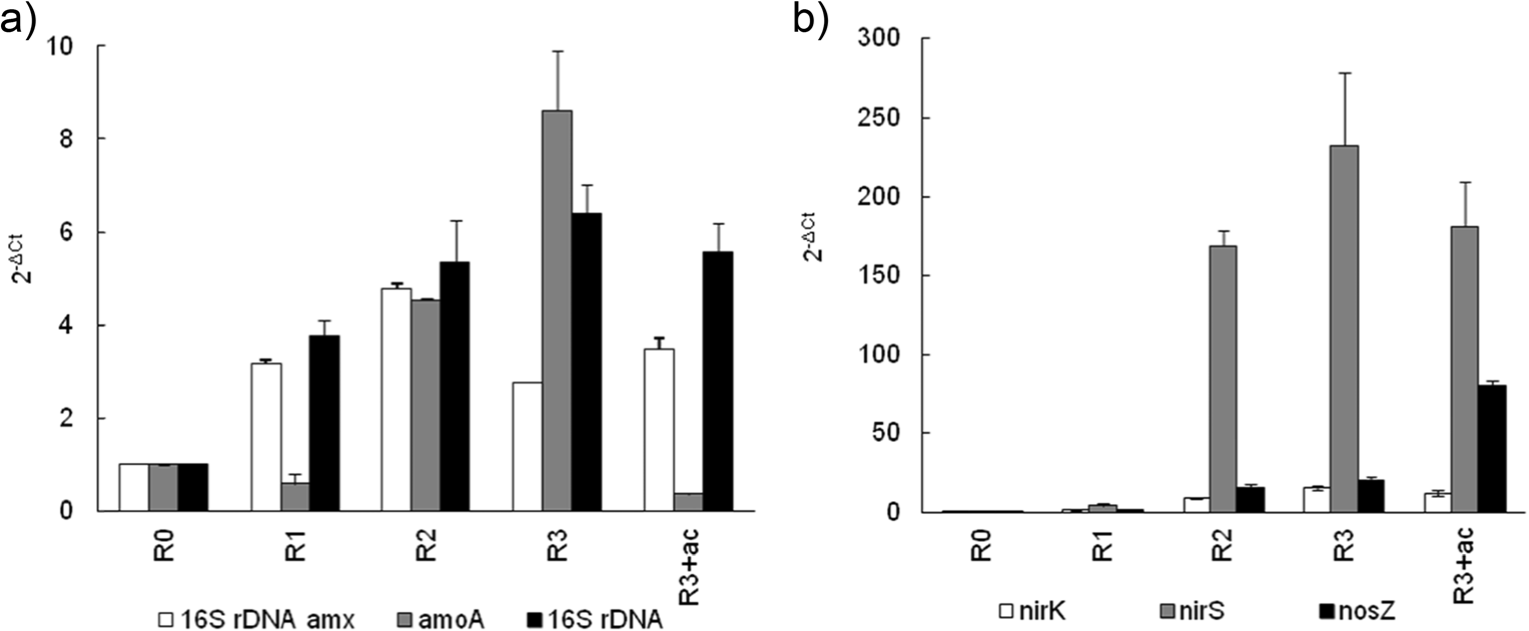
The relative number of copies of bacterial **a** 16S rDNA of Anammox bacteria (16S rDNA amx), *amoA* genes, and 16S rDNA and **b** *nirK*, *nirS*, and *nosZ* genes in the granules from the GSBRs

Following the introduction of non-aeration phases into the reactor cycle, the number of gene copies of bacterial 16S ribosomal DNA (rDNA) was four to six times higher than in the continuously aerated *R*_0_ (Fig. 3a). The number of 16S rDNA copies was positively correlated with the density of the granules (*R* = 0.99) and negatively with their diameter and mass (*R* = −0.97, *R* = −0.99).

There was no significant difference in the number of *amoA* gene copies in *R*_0_ and *R*_1_. When two or three non-aeration phases were introduced, the number of gene copies was 4.5 and 8.6 times higher, respectively, than in *R*_0_. The pulse feeding of acetate in the second non-aeration phase in *R*_3+ac_ decreased the number of *amoA* gene copies in the granules to about 0.5 value of *R*_0_. The introduction of non-aeration phases to the reactor cycle increased the copy number of 16S rDNA gene of the Anammox bacteria in the granules in all reactors; the highest increase (over fourfold) was noted in *R*_2_. The pulse feeding of acetate did not diminish the abundance of Anammox bacteria in the biomass.

The relative abundance of denitrifying genes in aerobic granules increased as the number of non-aeration phases in the GSBR cycle was increased (Fig. 3b). The number of *nirK* gene copies in the granules from *R*_3_ and *R*_3+ac_ was significantly higher than in *R*_0_ and *R*_1_. The numbers of *nirS* gene copies in *R*_1_, *R*_2_, *R*_3_, and *R*_3+ac_ were significantly 4, 169, 232, and 181 times higher, respectively, than in *R*_0_. The significantly highest number of *nosZ* gene copies was recorded in the granules from GSBR with acetate pulse feeding; it was over 80 times higher than in *R*_0_. Only the abundance of *nosZ* genes in the biomass was positively correlated with the efficiency of denitrification (*R* = 0.96) and nitrogen removal in the GSBRs (*R* = 0.98).

The most diverse AOB communities were in *R*_0_ and *R*_1_ (*H*′ > 3.0), and the diversity of AOB decreased with the increasing number of non-aeration phases in the GSBR cycle, reaching 1.9 ± 0.1 in *R*_3_. The diversity of bacteria with the *nirS* and *nirK* genes was similar in the biomass from all GSBRs, with *H*′ values of about 2.5 for *nirS* and from 2.5 ± 0.2 in *R*_0_ to 3.0 ± 0.2 in both *R*_2_ and *R*_3_ for *nirK*. Introduction of at least two non-aeration periods in the GSBR cycle significantly lowered the diversity of bacteria with the *nosZ* gene (*H*′ about 1.6 in *R*_2_, *R*_3_, and *R*_3+ac_) in comparison with the diversity in granules from *R*_0_ (*H*′ = 2.7 ± 0.3) and *R*_1_ (*H*′ = 3.0 ± 0.0).

**Fig. 4:**
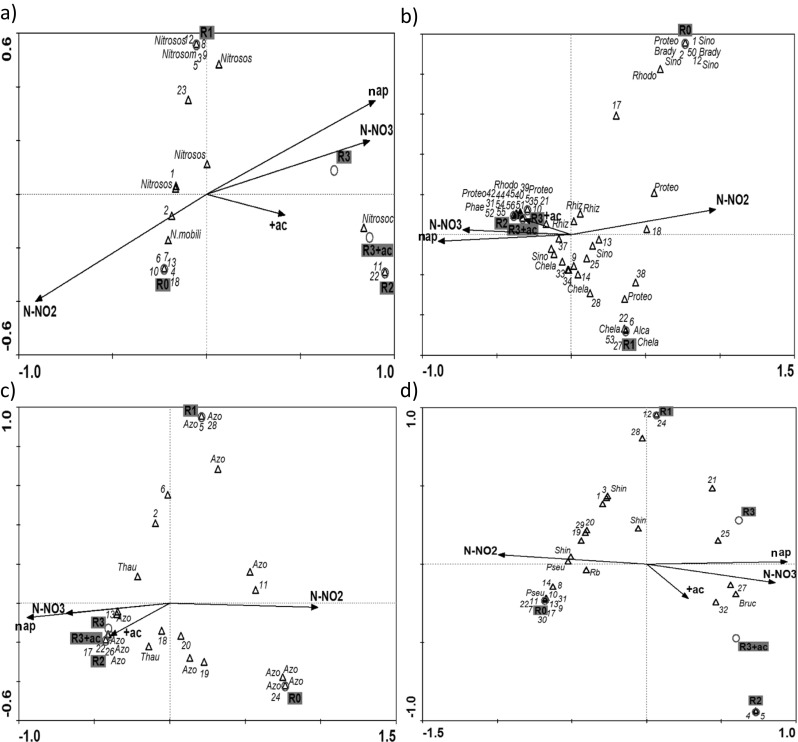
Triplots from a CCA of community of bacteria possessing **a** *amoA*, **b** *nirK*, **c** *nirS*, and **d** *nosZ* genes. Environmental variables are represented by *black arrows* (*N-NO*_*2*_ nitrite concentration in the effluent, *N-NO*_*3*_ nitrate concentration in the effluent, *+ac* acetate pulse feeding, *nap* the number of non-aeration phases in the GSBR cycle), samples by *gray circles*, and species by *black triangles*. The species are listed by letters and abbreviation and include *Nitrosococcus* (*Nitrosoc*), *Nitrosococcus mobilis* (*N. mobili*), *Nitrosomonas* sp. (*Nitrosom*), *Nitrosospira* sp. (*Nitrosos*), *Rhodobacter* sp. (*Rhodo*), *Alcaligenes faecalis* (*Alca*), *Rhizobium* sp. (*Rhiz*), *Bradyrhizobiaceae* (*Brady*), *Proteobacteria* (*Proteo*), *Chelativorans* sp. (*Chela*), *Sinorhizobium* sp. (*Sino*), *Phaeobacter gallaeciensis* (*Phae*), *Azoarcus tolulyticus* (*Azo*), *Thauera* sp. (*Thau*), *Shinella zoogloeoides* (*Shin*), *Brucella* sp. (*Bruc*), *Pseudomonas* sp. (*Pseu*), *Rhizobiales* sp. (*Rb*)

To connect operational variables with microbial structure of biomass, CCA was conducted (Fig. 4). In all GSBRs, species composition of granular sludge significantly influenced the nitrite concentration in the GSBR effluent; part of this nitrite remained in the GSBRs and was available for the microorganisms in the next cycle.

DGGE pattern characterizing AOB is shown in Fig. 2SM. Bands were cut out of the DGGE gel, sequenced, and analyzed phylogenetically (Fig. 3SM). The CCA showed that the analyzed environmental variables explained 83.7 % variability in the AOB community. *Nitrosococcus mobilis* (band 1A) was present only in constantly aerated *R*_0_ (Fig. 4a). In *R*_0_ and *R*_1_, *Nitrosospira* sp. occurred (bands 4A, 5A, and 6A) and most of the identified sequences grouped in the phylogenetic tree with the sequence of *Nitrosospira* sp. REGAU. With increasing number of non-aeration phases in the GSBR cycle, *Nitrosospira* sp. diminished and *Nitrosomonas* sp. occurred (bands 3A and 7A). Band 7A disappeared in the reactor with pulse feeding of acetate (*R*_3+ac_). *Nitrosococcus* sp. LT-3 (band 2A) was present in the biomass from GSBRs with at least two non-aeration phases in the cycle.

The results of PCR-DGGE for *nirK* gene are shown in Fig. 4SM, and phylogenetic affiliation of the obtained sequences is presented in Fig. 5SM. The location of samples and species of bacteria with nitrite reductases (both *nirK* and *nirS* gene, Fig. 4b, c), with respect to the *x*-axis, depended on the concentration of nitrite and nitrate in the effluents and the number of non-aeration phases. The CCA for *nirK* gene (Fig. 4b) showed that the environmental variables explained 82.3 % variations in the species structure of *nirK*-possessing bacteria in granules. Members of *Bradyrhizobiaceae* (bands 7K and 8K) occurred only in granules from the *R*_0_. *Alcaligenes faecalis* was present in *R*_1_ (band 5K), while *Chelativorans* sp. occurred in the reactors with at least one non-aeration phase (bands 11K, 15K, 17K, and 18K). *Phaeobacter gallaeciensis* (band 23K) and *Rhodobacter* sp. (band 4K) were present in the DGGE patterns characterizing granules from the GSBRs with at least two non-aeration phases in the cycle. *Rhizobium* sp. (e.g., bands 6K and 22K) were identified in all GSBRs. *Sinorhizobium* sp. occurred in the granules from all GSBRs (bands 9K and 16K), but some species (bands 3K, 13K, and 14K) were present only in the constantly aerated *R*_0_.

The results of PCR-DGGE for *nirS* gene are shown in Fig. 6SM, and phylogenetic affiliation of the obtained sequences is presented in Fig. 7SM. Analysis of environmental variables in the CCA explained 94.6 % variability in the community of bacteria possessing *nirS* gene. The community was significantly influenced by both nitrite and nitrate concentrations in the effluent (Fig. 4c). The majority of identified *nirS* sequences were most similar to the sequence of *Azoarcus tolulyticus*. These sequences were obtained from granules from all reactors. Similarly, *Thauera* sp. (bands 1S and 2S) were present in all GSBRs, though the intensity of band 1S rose with an increased number of non-aeration phases in the GSBR cycle.

The DGGE separation of PCR products obtained by using primers that recognize sequences of *nosZ* gene is shown in Fig. 8SM and the phylogenetic analysis of the obtained sequences in Fig. 9SM. The CCA showed that the analyzed environmental variables explained 83.4 % variability in the community of bacteria with the nitrous oxide reductase gene. *Shinella zoogloeoides* (bands 1Z and 3Z) and *Pseudomonas* sp. (bands 4Z and 7Z) were mostly identified in *R*_0_ and *R*_1_ (Fig. 4d). However, band 6Z with a sequence similar to *Shinella zoogloeoides* characterized granules from all GSBRs. Introduction of at least one non-aeration phase in the GSBR cycle resulted in the occurrence of *Brucella* sp. (band 2Z).

## Discussion

The growth of desirable bacteria in the structure of aerobic granules can be supported by controlling the operational parameters of the GSBR. The present study aimed to investigate how non-aeration phases and acetate pulse feeding in the GSBR cycle affect the physicochemical and microbial properties of aerobic granules and the efficiency of nitrogen removal from high-nitrogen wastewater with a low COD/N ratio. For insight into the main mechanisms of nitrogen conversion, the species composition and abundance of the nitrogen-converting communities in the granules were analyzed on the basis of 16S rDNA and functional genes.

Both non-aeration phases and acetate pulse feeding during the GSBR cycle changed the characteristics of the biomass, i.e., biomass concentration, granule diameters, EPS content, or hydrophobicity. These characteristics determined the microorganisms that were present and the manner in which nitrogen was converted in the granules’ structure. In the study, high biomass concentrations were obtained in all GSBRs, and the granules were characterized by very good settling properties (SVI below 37 mL/g MLSS). It was, however, observed that the concentrations of total suspended solids in the effluents from all GSBRs were high (0.36 ± 0.06–0.46 ± 0.22 g TSS/L). These phenomena may be observed in aerobic granule systems operated at very short settling times per cycle; in this study, it was 5 min. Such a short sedimentation time ensures removal of the biomass that does not settle well and exerts a very high pressure for efficient granulation. In a full-scale treatment line, the settling time should be therefore lengthened or these suspended solids should be removed with a secondary clarifier or a membrane module. Granule diameter is one of the most important morphological feature that is influenced by operational parameters and translates to the efficiency of nitrogen removal. Reduction of the diameter of the granules from about 0.250 to 0.125 mm by increasing the intensity of mixing in the reactor decreased denitrification efficiency by 60 % (Wan and Sperandio 2009). The present study found that the most nitrogen was removed when the granules predominately had diameters of 0.25–1.0 mm.

The EPS content in granules was reduced by lengthening of non-aeration periods in the GSBR cycle. This explains a decrease in biomass concentrations in the experimental GSBRs from 10.8 ± 2.2 g MLSS/L in *R*_1_ to 6.6 ± 1.5 g MLSS/L in *R*_3_. The low content of EPS in biomass from *R*_2_ and *R*_3_ can also result from the higher abundance of aerobic AOB that do not produce extracellular polysaccharides (Tsuneda et al. 2001). Availability of easily biodegradable organics due to acetate pulse feeding increased EPS content in granules from *R*_3+ac_ to about 0.4 g/g MLSS in comparison with 0.17 g/g MLSS in the biomass from *R*_3_. These EPS could be available for the bacteria providing electron donors for denitrification and supporting the most efficient nitrogen removal in *R*_3+ac_.

The efficiency of ammonia removal by the aerobic granules exceeded 90 %. In our studies, there was no risk of ammonia stripping since it occurs at pH 10.8–11.5, which is much higher than in our GSBRs. After the addition of non-aeration phases into the cycle, the rate of ammonia removal slightly decreased, but in each of the GSBRs, ammonia was removed in less than 5 h of the 8-h cycle. It allowed the safe operation of the system at safety coefficient ca. 35 % and the balancing of the cycle in case of variations in the pollutant load in the influent. The short time for ammonia depletion shows that it is also possible to shorten aeration, thus saving energy during the treatment of digester supernatant.

Both aerobic and anaerobic ammonia oxidizers were present in the biomass. AOB abundance increased with a greater number of non-aeration phases in the cycle but diminished after pulse feeding of acetate. Addition of external organics, on the other hand, did not significantly diminish the abundance of Anammox bacteria, which remained in the biomass from *R*_3+ac_ and maintained efficient ammonia removal. Anammox bacteria are facultative chemoorganotrophs and can use pyruvate, acetate, and propionate as their substrates (Strous et al. 2006). They can use ammonia as an electron donor and cometabolize organics that are present in the environment (Kartal et al. 2007).

In our study, nitrite accumulated in all GSBRs as a result of partial nitrification, which is expected with ammonia concentrations of 600–800 mg/L in the influent (Ruiz et al. 2003). However, it was observed that nitrite accumulation decreased as the number of non-aeration phases in the cycle was increased. With one non-aeration phase in the cycle, nitrite concentration was about five times higher than in the reactors with at least two non-aeration phases in the cycle. According to Anthonisen et al. (1976) and Chang et al. (2002), inhibition of nitrite-oxidizing bacteria (NOB) can be caused by free nitrous acid (FNA) and free ammonia (FA). In our studies, calculated concentrations of free nitrous acid (FNA) were below 0.0003 mg HNO_2_-N/L, and compared with the values of 0.06–0.85 mg HNO_2_-N/L, proposed by (Anthonisen et al. 1976), they cannot be regarded as inhibitory concentrations.

In the present study, the concentration of FA was 53–91 mg/L in the initial hours of the cycle, so the inhibition may have occurred at this time. However, from about the fourth hour of the cycle, the concentration of ammonia dropped to such levels that there was no longer FA inhibition to NOB, but still, the accumulation of nitrite occurred. It can be suspected that low formation of nitrate in *R*_0_ and *R*_1_ resulted from biomass morphology. In *R*_0_ and *R*_1_, granules had about two times greater diameters than in *R*_2_, *R*_3_, and *R*_3+ac_. Granule diameters affect the distribution of AOB and NOB in the biomass (Bin et al. 2011). In small and medium granules (<0.6 mm), oxygen mass transfer is not restricted. Larger granules have smaller aerobic volume fraction, and inhibition of NOB growth occurs. AOB can survive under the conditions of low DO since they have a higher affinity toward oxygen than NOB (Ma et al. 2009; Bin et al. 2011). The decrease in nitrite accumulation and the parallel growth in nitrate concentrations in *R*_2_ can result from an increased presence of Anammox microorganisms, which use nitrite as a substrate for growth. Intermittent oxic/anoxic conditions support the growth in granules of fast-growing nitrite-oxidizing bacteria, e.g., *Nitrobacter* sp. (Dytczak et al. 2008), which could also play an important role in efficient nitrite removal.

In the present experiment, the pulse feeding of acetate significantly increased nitrite concentration in the effluent. Accumulation of nitrite may have resulted from faster reduction of nitrate than nitrite at a high concentration of electron donors in the environment (Pan et al. 2012). Nitrate-respiring and denitrifying bacteria coexist in granules (Glass and Silverstein 1999). Nitrate-respiring bacteria have an advantage over denitrifying bacteria in a mixed population because the cell yield for nitrate reduction by nitrate-respiring bacteria is three times higher than that of denitrifying bacteria (Turk and Mavinic 1987).

The availability of organics had greater influence on the denitrification efficiency in wastewater with a low COD/N ratio than the number of non-aeration phases in the GSBR cycle. The presence of one non-aeration phase significantly improved denitrification, compared to the constantly aerated control reactor. However, the introduction of two and three non-aeration phases did not further increase the efficiency of denitrification, and the overall nitrogen removal efficiency did not exceed 40 %. The introduction of non-aeration phases, however, allowed maintenance of effective full nitrification, improving the nitrate to nitrite ratio in the effluent. It should also be stressed that the operation of the reactor with a higher number of non-aeration phases decreases aeration costs, while maintaining the quality of the effluent comparable to GSBR operated with one non-aeration phase.

The pulse feeding of acetate in the GSBR significantly increased the efficiency of denitrification to 60 % and gave a total efficiency of nitrogen removal above 70 %. In a constantly aerated granular sludge reactor treating high-nitrogen wastewater, organics were stored in granules in the form of poly-β-hydroxybutyrate (Cydzik-Kwiatkowska et al. 2012) and the storage of polyhydroxyalkanoates is favored by intermittent anoxic and aerobic conditions (Third et al. 2003). It can be, therefore, supposed that some of the pulse-fed organics were accumulated in bacterial cells and served as carbon sources for the denitrification in the third non-aeration period and the final aerobic part of the GSBR cycle, which was indicated by the decrease in oxidized nitrogen forms.

There was a dynamic correlation between the operational parameters, microbial structure, and resulting morphology of granules. The number of gene copies of bacterial 16S rDNA in biomass gradually increased following the introduction of successive non-aeration phases into the reactor cycle. Facultative anaerobes, whose growth was promoted by non-aeration periods, tend to have more than five copies of 16S rRNA in their genomes (Větrovský and Baldrian 2013). Moreover, the decrease in granule diameter meant that oxygen and substrates more easily diffused in granule structure, so the whole granule volume was inhabited by living bacteria.

The introduction of non-aeration phases in the GSBR cycle increased the number of denitrifying bacteria in granules. However, out of the three investigated denitrification genes (*nirK*, *nirS*, and *nosZ*), only the abundance of bacterial *nosZ* was associated with the efficiency of denitrification and nitrogen removal. The results point to a low sensitivity of nitrite reducers to operational parameters of GSBR. Low correlation between nitrogen removal and the abundance of bacterial *nirK* and *nirS* genes can be explained by a wide distribution of these genes in genomes of other microorganisms, e.g., fungi (Zumft 1997). This ensures stable conversion of NO_2_^−^ to NO, even at lower abundance of bacteria possessing nitrite reductase genes. The pulse feeding of acetate significantly increased the number of *nosZ*-possessing bacteria able to denitrify to N_2_. Significant reduction of diversity of *nosZ* genes with the introduction of non-aeration phases was probably due to better oxygen diffusion in granules with smaller diameters; nitrous oxide reductase is a denitrification enzyme that is the most sensitive to the presence of oxygen in the environment. Changes in diversity of denitrifiers in granules did not affect nitrogen removal.

The number of Anammox bacteria in granules increased with the introduction of non-aeration phases; however, the introduction of third non-aeration phase caused the decrease of their number. There could be two reasons for this. The first is the competition for a substrate (NO_2_^−^) with denitrifying bacteria; the number of denitrifiers possessing *nirS* gene was the highest in the GSBR with three non-aeration phases. Another reason is that a decrease in diameter of the granules with the increase of the number of non-aeration phases reduced the anaerobic zone inside the granules thus restricting the environment for Anammox bacteria.

*Azoarcus* sp., *Thauera* sp., *Rhizobium* sp., and *Shinella* sp., which are common denitrifiers in wastewater treatment systems with activated sludge (Wang et al. 2014b), were core genera of denitrifiers in granules independent of the GSBR operation. These bacteria are able to metabolize various organic compounds, e.g., amino acids and aromatic compounds, under both aerobic and non-aeration conditions. *Thauera* sp. can use a wider range of substrates than *Azoarcus* sp. (Mechichi et al. 2002; Thomsen et al. 2007) and often predominates in systems with long SRT, such as granular or fluidized bed reactors (Araki et al. 2006; Zhao et al. 2013). The presence of *Thauera* sp. supported granulation since this genus produces EPS responsible for the cell aggregation (Zhao et al. 2013). *Rhizobium* sp. and *Shinella zoogloeoides* (formerly *Zoogloea ramigera*), that occurred in granules from all GSBRs, produce polysaccharides that constitute part of the EPS and also support granulation (Pal and Paul 2008).

The study identified bacteria sensitive to both lengthening of non-aeration periods in the GSBR cycle and organics pulse feeding. *Pseudomonas* sp. and *A. faecalis* are aerobic denitrifiers, able to perform heterotrophic nitrification and ammonia removal without accumulation of nitrite and nitrate (Kathiravan and Krishnani 2013, 2014), which were present in granules from *R*_0_ and *R*_1_; a greater number of non-aeration phases inhibited their growth. *A. faecalis* NR I is also able to remove nitrogen by the oxidation of ammonia nitrogen to N_2_ with hydroxylamine as the only intermediate product (Zhao et al. 2012). In the present study, bacteria of the genus *Pseudomonas* were identified on the basis of *nosZ* gene sequence analysis and *A. faecalis* on the basis of *nirK* gene sequence. This supports the conclusion of Kathiravan and Krishnani (2013) that due to the absence of *amoA*, *napA*, *narG*, *nirS*, or *qnorB* in the genomes of heterotrophic nitrifiers/aerobic denitrifiers, studies on these bacteria should focus on *nosZ* and *nirK* genes.

AOB diversity diminished as the number of non-aeration phases increased, and the species structure shifted from *N. mobilis* and *Nitrosospira* sp. in *R*_0_ to *Nitrosospira* sp. and *Nitrosomonas* sp. in reactors with at least one non-aeration phase and *Nitrosococcus* sp. LT-3 in GSBRs with at least two non-aeration phases. Denitrifying bacteria of the genera *Chelativorans* and *Brucella* sp. were present in the GSBRs operated with at least one non-aeration phase in the cycle, but *Brucella* sp. did not disappear after pulse feeding of acetate. *Brucella* sp. have a control system that, in response to anaerobic conditions and organics availability, induces the expression of cytochrome oxidase and denitrification genes which favors their presence in aerobic granules from GSBRs operated with multiple non-aeration periods (Carrica et al. 2013). The presence of at least two non-aeration phases stimulated growth of *Phaeobacter gallaciencis* and *Rhodobacter* sp. Species of the genus *Rhodobacter* have been found in various types of wastewater treatment plants, including industrial plants.

## Conclusions

A 70 % efficiency of N removal from real digester supernatant was obtained in a one-stage GSBR with three non-aeration phases and pulse feeding of acetate in the cycle. In this technological solution, the shortened aeration time due to the presence of non-aeration periods in the GSBR cycle saves energy concerned with wastewater aeration. The technological and molecular analyses provided conclusions about the ecology, microbial composition, and EPS production in complex biocenoses of aerobic granules. The introduction of three non-aeration phases in the GSBR cycle lowered the diversity of both AOB and *nosZ*-possessing denitrifiers; however, this lowered diversity did not worsen either ammonia oxidation or denitrification efficiencies. The acetate pulse feeding in the GSBR cycle supported the growth of denitrifiers possessing the *nosZ* gene, thus promoting denitrification to N_2_.

## Electronic supplementary material

ESM 1 (PDF 272 kb)
