# Supplementary material for: Microbial structure and nitrogen compound conversions in aerobic granular sludge reactors with non-aeration phases and acetate pulse feeding
Source: Environ Sci Pollut Res Int. 2016 Sep 23;23(24):24857–70. doi: 10.1007/s11356-016-7709-7 (PMC5124037; doi:10.1007/s11356-016-7709-7)
Supplement: Supplementary file 1 — (PDF 272 kb) [file 11356_2016_7709_MOESM1_ESM.pdf]

Acetate pulse-feeding and the number of non-aeration phases affect nitrogen-converting communities in aerobic granules treating high-ammonia reject water

Agnieszka Cydzik-Kwiatkowska*, Paulina Rusanowska, Magdalena Zielińska, Katarzyna Bernat, Irena Wojnowska-Baryła

University of Warmia and Mazury in Olsztyn, Słoneczna 45G, 10-709 Olsztyn, Poland

* corresponding author: agnieszka.cydzik@uwm.edu.pl, tel. +48 89 5234194, fax +48 89 5234131

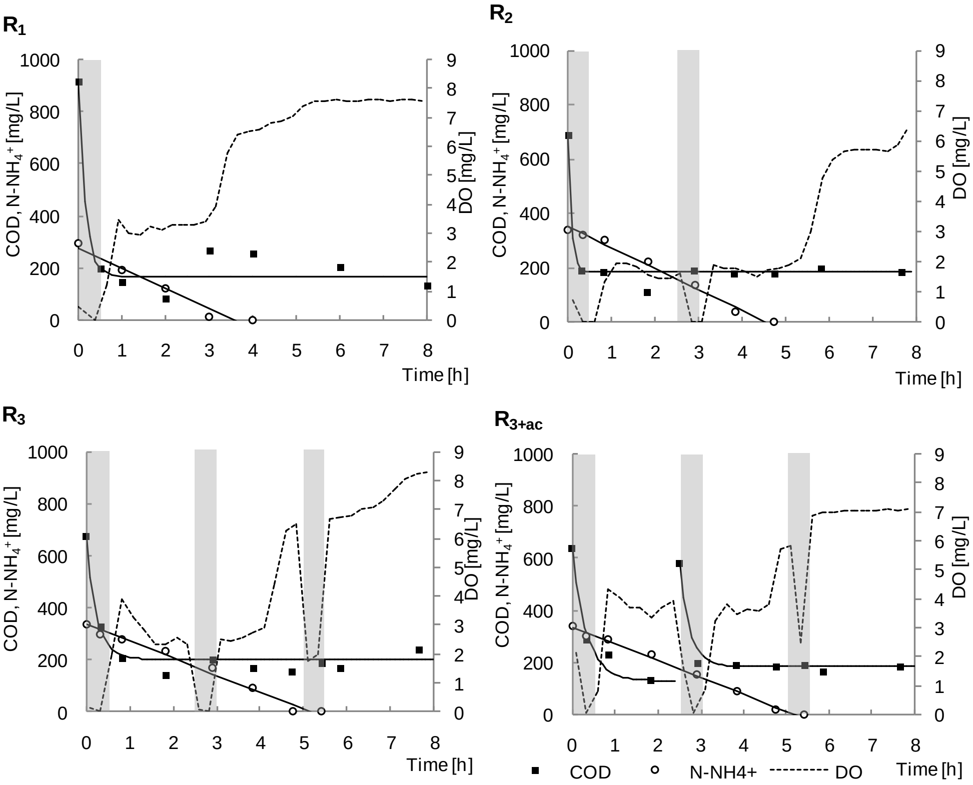

Fig. 1SM Changes in the concentrations of ammonia and organic compounds (COD) and dissolved oxygen (DO) during the cycle of the GSBRs; grey shading indicates non-aeration phases; constant lines indicate zeroth and first order kinetics for ammonia and COD experimental data, respectively

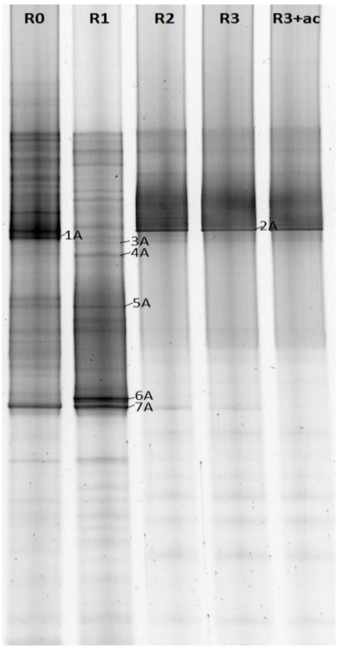

Fig. 2SM Separation of PCR products obtained using primers amoA1 and amoA2R, in denaturing gel (PCR-DGGE); in the picture sequenced bands are selected; in the electrophoretic paths the following samples are given: R_0, R_1, R_2, R_3, R_{3+ac}; gel was stained with SYBRGold (Molecular Probes); in the picture colors were reversed

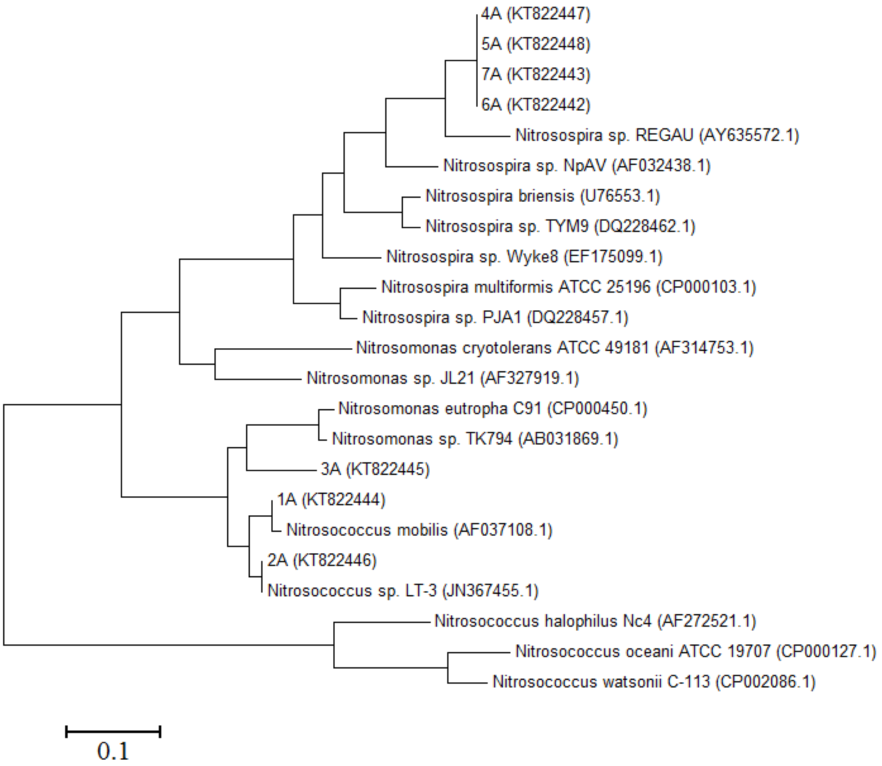

Fig. 3SM Phylogenetic tree showing the similarity between the *amoA* gene sequences obtained in the study and reference sequences from FunGene and Gene Bank (accession numbers in brackets). Evolutionary history concluded using the maximum likelihood method based on the Tamura-Nei model (Tamura and Nei, 1993).

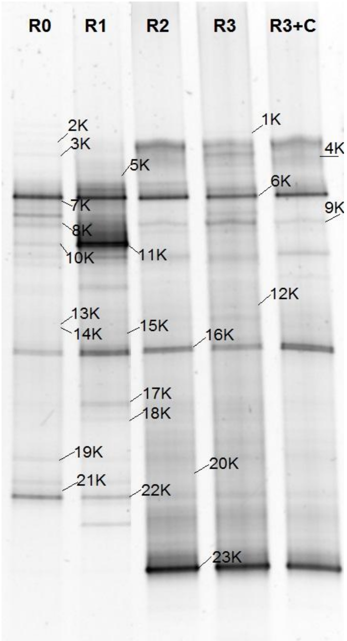

Fig. 4SM Separation of PCR products obtained using primers F1aCu/R3Cu, in denaturing gel (PCR-DGGE); in the picture sequenced bands are selected; in the electrophoretic paths the following samples are given: R_0, R_1, R_2, R_3, R_{3+ac}; gel was stained with SYBRGold (Molecular Probes); in the picture colors were reversed

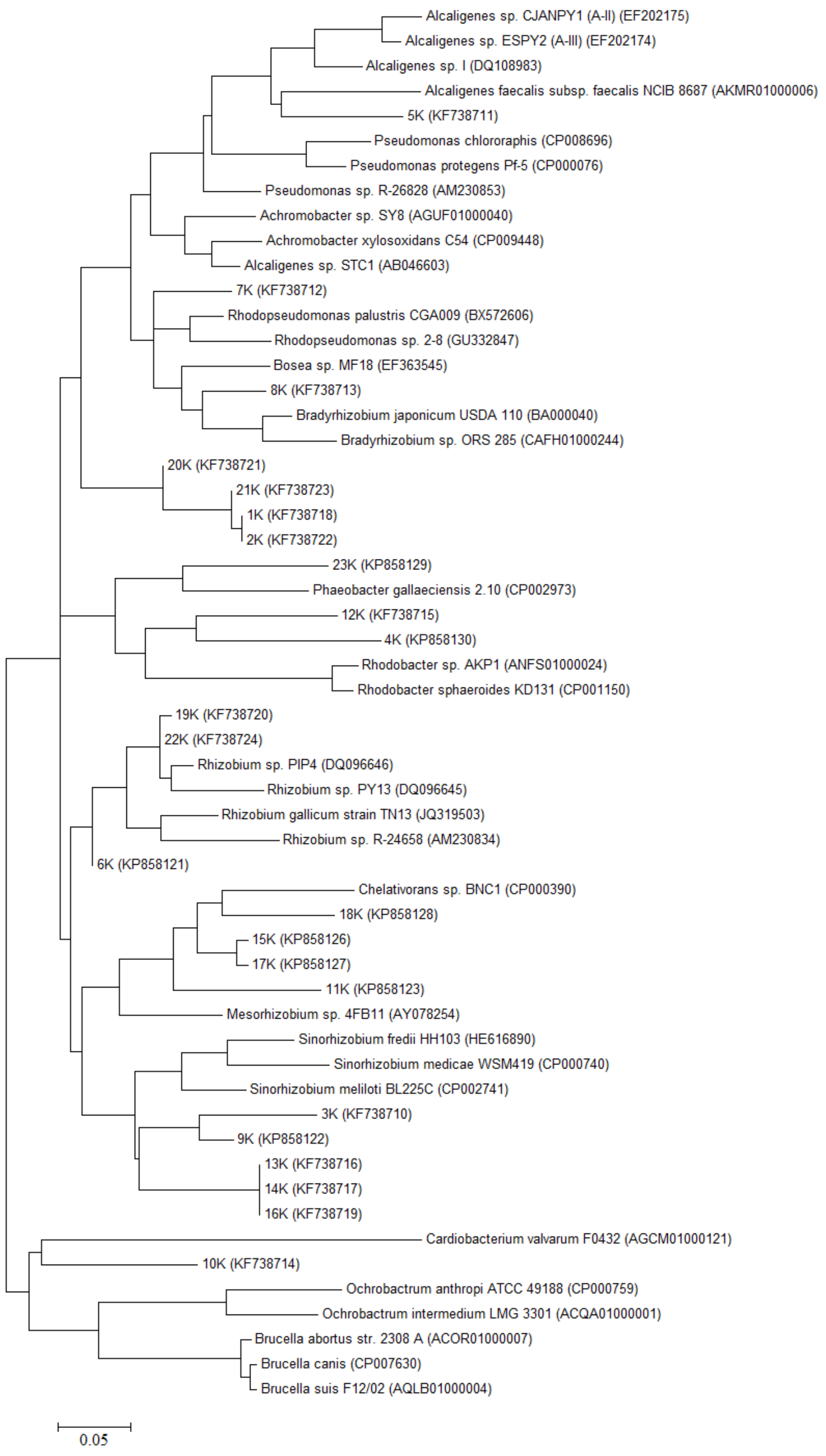

Fig. 5SM Phylogenetic tree showing the similarity between the *nirK* gene sequences obtained in the study and reference sequences from FunGene and Gene Bank (accession numbers in brackets). Evolutionary history concluded using the maximum likelihood method based on the Tamura-Nei model (Tamura and Nei, 1993).

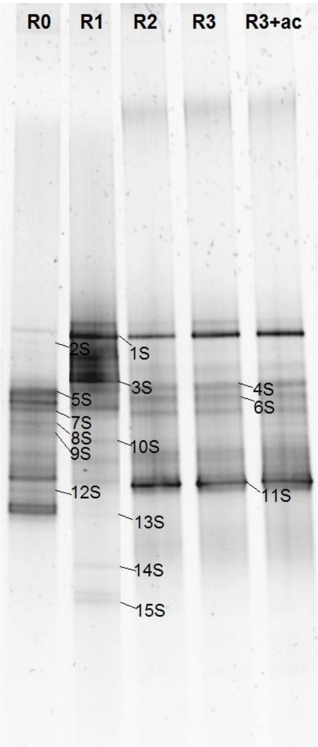

Fig. 6SM Separation of PCR products obtained using primers cd3aF/R3cd, in denaturing gel (PCR-DGGE); in the picture sequenced bands are selected; in the electrophoretic paths the following samples are given: R_0, R_1, R_2, R_3, R_{3+ac}; gel was stained with SYBRGold (Molecular Probes); in the picture colors were reversed

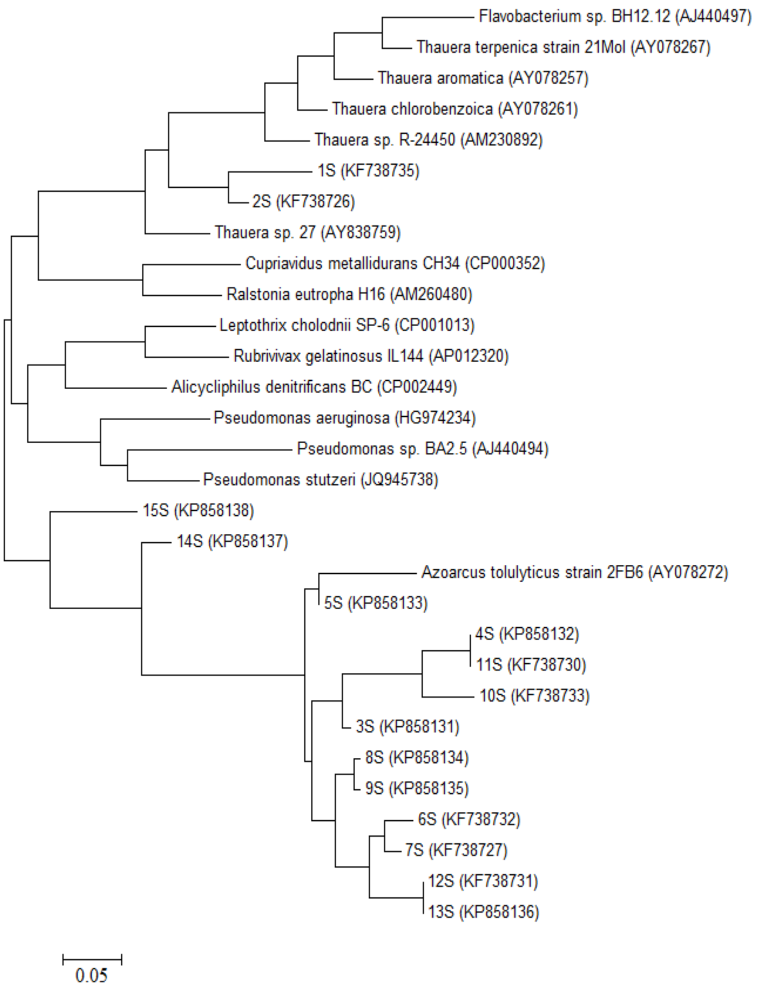

Fig. 7SM Phylogenetic tree showing the similarity between the *nirS* gene sequences obtained in the study and reference sequences from FunGene and Gene Bank (accession numbers in brackets). Evolutionary history concluded using the maximum likelihood method based on the Tamura-Nei model (Tamura and Nei, 1993).

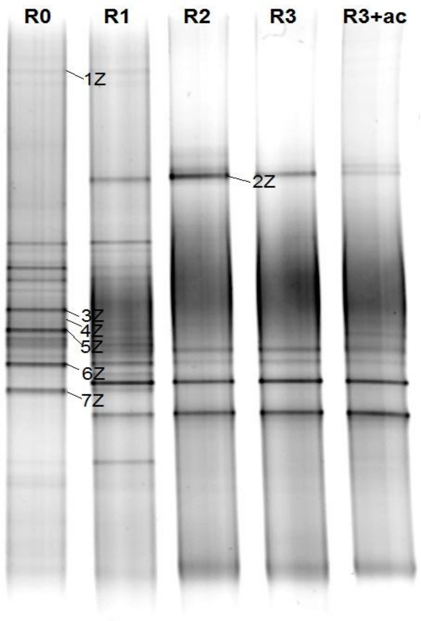

Fig. 8SM Separation of PCR products obtained using primers NosZ-F/NosZ1622-R, in denaturing gel (PCR-DGGE); in the picture sequenced bands are selected; in the electrophoretic paths the following samples are given: R_0, R_1, R_2, R_3, R_{3+ac}; gel was stained with SYBRGold (Molecular Probes); in the picture colors were reversed

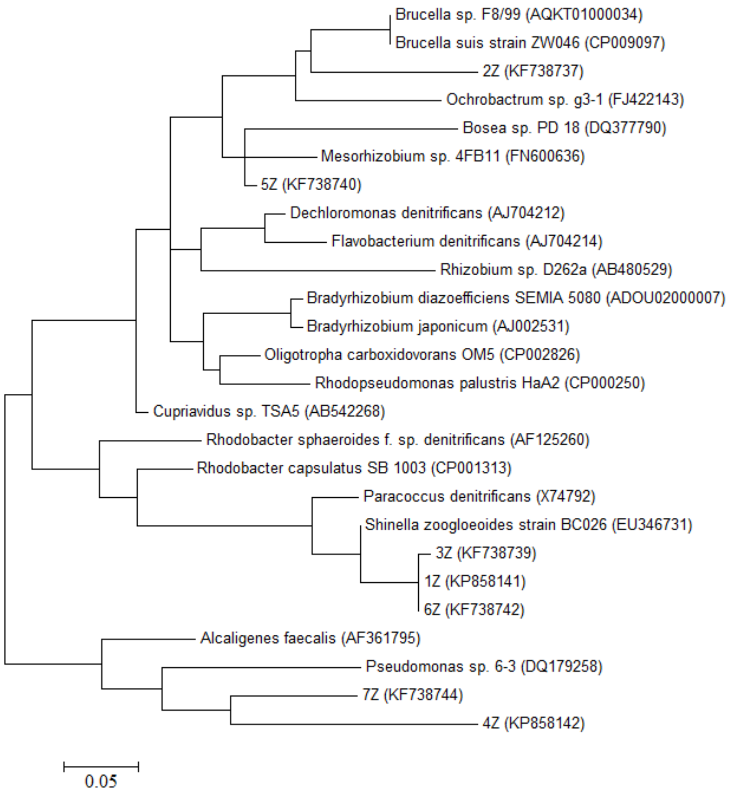

Fig. 9SM Phylogenetic tree showing the similarity between the *nosZ* gene sequences obtained in the study and reference sequences from FunGene and Gene Bank (accession numbers in brackets). Evolutionary history concluded using the maximum likelihood method based on the Tamura-Nei model (Tamura and Nei, 1993).
